# Polysubstance Use among Patients Enrolling in Methadone Maintenance Treatment Program in a Vietnam Province with Drug-Driven HIV Epidemic

**DOI:** 10.3390/ijerph16183277

**Published:** 2019-09-06

**Authors:** Tuan Anh Le, Duyen T. T. Pham, Travis T. C. Quek, Giang Thu Vu, Chi Linh Hoang, Tung Thanh Tran, Cuong Tat Nguyen, Ninh Hai Thi Tran, Quan Hoang Vuong, Tung Hoang Tran, Bach Xuan Tran, Carl A. Latkin, Cyrus S. H. Ho, Roger C. M. Ho

**Affiliations:** 1 National Institute of Hygiene and Epidemiology, Hanoi 100000, Vietnam (T.A.L.) (D.T.T.P.); 2 Institute for Preventive Medicine and Public Health, Hanoi Medical University, Hanoi 100000, Vietnam; 3 Department of Psychological Medicine, Yong Loo Lin School of Medicine, National University of Singapore, Singapore 119074, Singapore (T.T.C.Q.) (R.C.M.H.); 4 Center of Excellence in Evidence-Based Medicine, Nguyen Tat Thanh University, Ho Chi Minh City 700000, Vietnam (G.T.V.) (T.T.T.); 5 Center of Excellence in Behavior Medicine, Nguyen Tat Thanh University, Ho Chi Minh City 700000, Vietnam; 6 Institute for Global Health Innovations, Duy Tan University, Da Nang 550000, Vietnam; 7 National Hospital for Tropical Diseases, Hanoi 100000, Vietnam; 8 Centre for Interdisciplinary Social Research, Phenikaa University, Yen Nghia, Ha Dong, Hanoi 100803, Vietnam; 9 Faculty of Economics and Finance, Phenikaa University, Yen Nghia, Ha Dong 100803, Hanoi, Vietnam; 10 Institute of Orthopaedic and Trauma Surgery, Vietnam—Germany Hospital, Hanoi 100000, Vietnam; 11 Bloomberg School of Public Health, Johns Hopkins University, Baltimore, MD 21205, USA; 12 Department of Psychological Medicine, National University Hospital, Singapore 119074, Singapore; 13 Institute for Health Innovation and Technology (iHealthtech), National University of Singapore, Singapore 119074, Singapore

**Keywords:** MMT, polysubstance use, Vietnam, HIV

## Abstract

Methadone maintenance treatment (MMT) has been scaled up significantly in recent years. This study aimed to investigate the pattern of polysubstance use in 395 MMT patients and its contextualized associated factors. A cross-sectional study was performed in three outpatient MMT clinics in Nam Dinh Province. Multivariate Poisson regression was used to identify factors associated with polysubstance use status. The mean MMT duration and the current MMT dose was 3.3 years and 69.2 mg, respectively. Among participants, 24.8% reported daily alcohol use, 68.6% smoked regularly, and 6% used illicit drugs. Peer pressure and MMT suboptimal adherence were found to associate with continual usage of drugs (47.8%). Participants who lived with a spouse/partner, were self-employed, and smoked were more likely to drink alcohol. Those who drink were also more likely to smoke, and vice versa. Recommendations for policymakers include community-based education and promotional programs aiming to decrease substance usage in the community as well as encouraging and supporting the private health sector in establishing private MMT services and clinics. Further longitudinal studies on polysubstance usage among MMT patients should also be conducted.

## 1. Introduction

The Vietnamese Government estimates the number of people who inject drugs (PWIDs) in the country to be around 271,000 [1], of which 85% are heroin users. This population has been regarded as the key at-risk population for HIV infection in Vietnam, with HIV prevalence reported to be at 14%, higher than that of other at-risk populations such as sex workers (3.7%) and men who have sex with men (12.2%) [2]. Another report indicated that PWIDs accounted for 60% of newly HIV-infected cases in Vietnam [3]. Thus, interventions targeting this group would benefit the fight against the HIV/AIDS epidemic in the country. Among such interventions, while buprenorphine has been recently piloted for its feasibility and cost-effectiveness, methadone maintenance treatment (MMT) has been found to be one of the most cost-effective methods to evidently reduce the use of opioids among those enrolled in the program in a number of countries globally [4,5,6,7,8]. The Government of Vietnam piloted the program in Hai Phong and Ho Chi Minh in 2008 [9] and has been scaling up nationwide after the success of the pilot [5]. By March 2017, 51,318 drug users had been provided MMT by 280 methadone clinics in 63 cities and all provinces of Vietnam [8].

Polysubstance use or substance use disorder is a general term for a combination of several drugs for therapeutic or alternative purposes over a period of time [10]. It is widely acknowledged that the use multiple substances have increased recently [10]. Previous works have reported that the combination of opioids, for example, heroin with benzodiazepine, raises the levels of toxicity and overdose death [11]. In Vietnam, heroin has been known as the most commonly used drug, with the proportion of drug users using heroin ranging from 65% to 85% [12,13]. Heroin users have reported that they consumed heroin concurrently with other drugs, including crystal methamphetamine (15%), marijuana (7.0%), and amphetamines (2.8%) [14]. The combination of different drugs might be attributed to the purpose of increasing excitement, which is highly enjoyed by drug users [15].

In a similar vein, research has shown that concurrent use of other substances is not uncommon among patients registering for MMT programs [16,17,18]. A study of 434 patients in Ukraine receiving opioid agonist treatment found that 23% reported concurrent drug use in the previous 30 days [19]. In 53 stabilized patients undergoing MMT in Italy, almost 60% concurrently used cocaine, alcohol, or both [20]. Among 103 patients undergoing MMT in New York, USA, 40% used cocaine, 43% used alcohol, 19% used sedatives, 3% used hallucinogens, and 32% had used cannabis in the previous 30 days [21]. In Vietnam, a previous study revealed that while there were only 14.4% of patients testing positive for heroin urine samples [22], the prevalence of smoking and frequent alcohol drinking among MMT patients was 87.3% and 29.6%, respectively [23]. Another study on MMT in mountainous areas found the prevalence of concurrent opioid use to be 13.4% [24]. Hazardous alcohol consumption, smoking, and use of other substances have been found to have a significant impact on the long-term efficacy of MMT [25]. 

Many factors believed to be involved in the concurrent use of opioids in MMT patients have been elucidated. Age of onset of opioid abuse [26], frequency of opioid use [27], peer pressure [27,28], living with family members who use heroin [29], and concomitant antiretroviral and tuberculosis treatment [27] have all been found to be positive predictors of concurrent opioid use in MMT patients. 

In this study, we sought to contribute to the abundant MMT literature by examining polysubstance uses, which included both illicit drugs and drinking/smoking, in a pool of participants attending public and private MMT clinics in Nam Dinh, Vietnam, one of the areas with the highest number of opioid users. The inclusion of a private clinic is significant, as it is the first fully functioning private MMT clinic in Vietnam and has the potential to shoulder the burden of public clinics in providing MMT services in an era of shrinking foreign aid for programs to combat HIV/AIDS and drug use. In addition, as the prevalence of substance use as well as its determinants vary depending on specific settings, more contextualized factors should be thoroughly examined in order to develop robust interventions for improving the quality of care to and health of MMT patients.

## 2. Materials and Methods 

### 2.1. Study Design and Participants

From January to September 2018, we conducted a cross-sectional study in Nam Dinh Province. Nam Dinh is one of the largest settings offering care services for HIV/AIDS in the north of Vietnam. The study settings were three outpatient clinics, including the Giao Thuy district health center, the Dai Dong private health facility, and the Giao Thuy Center for Social Evils Prevention. These three clinics all have at least two years of experience in providing MMT services and are in accordance with the official guidelines of the Ministry of Health of Vietnam. 

Take-all sampling in the private health center and convenience sampling in the two public health centers were applied to recruit respondents for the study. All the respondents who met the inclusion criteria were invited to enroll to our study [30]. The inclusion criteria for respondents were (1) aged at least 18 years, (2) using MMT services from clinics mentioned above, and (3) willing to participate in the study by signing the informed consent form. Participants who suffered from serious illnesses or did not have the capacity to answer questions from data collectors were excluded from the recruitment process. A total of 395 participants agreed to participate in the study. The percentage of patients in each health facility who participated in the study was 100% (Dai Dong private health facility), 70.4% (Giao Thuy district health center), and 47.2% (Giao Thuy Center for Social Evils Prevention).

### 2.2. Measure and Instruments

Twenty-minute face-to-face interviews were conducted in a small counseling room to ensure privacy and confidentiality. Before answering the questionnaire, informed consent was obtained. The interviewers were well-trained researchers. Health staff from the MMT study settings were not invited to the interviews to avoid social desirability bias. Our research members invited participants to join the study when they received medication or counseling services. Eligible participants were defined based on feedback from the medical staff. A pilot survey was carried out among 20 participants of different ages, genders, and occupations. A structured questionnaire was developed to obtain the following information:

- Socioeconomic characteristics

Participants self-reported their information about age, education, marital status, occupation, and monthly family income.

- MMT-service-related characteristics

Participants’ time of MMT treatment was self-reported, and current MMT dose was extracted from the medical report. Participants self-reported their MMT adherence using a Likert scale (five options from “Very good” to “Very bad”). In addition, a 100-point visual analog scale (VAS) was applied to rate their adherence, with the score ranging from 0 “completely nonadherent” to 100 “completely adherent”. The patient was classified as optimal adherence if he assessed his MMT adherence at 100 points, and 0–99 points was defined as suboptimal.

- Health risk behavior

Participants self-reported their frequency of drinking alcohol and smoking tobacco. Regarding drug use, date of initially using drugs, whether they inject drugs, and currently using drugs or not were also examined. If they answered “Yes” to the question on current use, we asked them about the frequency, the main reasons for continuous drug use, and the amount of money spent on drugs. Based on alcohol, tobacco, and drug usage, we divided the sample into three levels of polysubstance use groups: none, using one substance, using two or more types of substances. 

### 2.3. Statistical Analysis

STATA version 12 (Stata Corp. LP, College Station, United States of America) was used for data analysis. Chi-squared and Kruskal–Wallis tests were used for analyzing the demographic characteristics of the participants as well as MMT-related characteristics and health risk behaviors. We also used multivariate logistic regression to find associated factors with alcohol, tobacco, and illicit drug usage. Multivariate Poisson regression was used to find factors related to polysubstance use status. A forward stepwise selection strategy with a threshold of 0.2 was applied to remove insignificant factors. A *p*-value of <0.05 was considered as statistically significant.

### 2.4. Ethical Approval

The study protocol was reviewed and granted ethics approval by the Institutional Review Board of the National Institute of Hygiene and Epidemiology (code: 726/QDVSDTTU).

## 3. Results

Table 1 describes the socioeconomic information of the participants in this study. The majority of participants had a secondary school education or above (83.3%). More than two-thirds of the respondents lived with a spouse/partner (77%). About one-third of the participants were self-employed (35.2%), followed by blue-collar workers/farmers (23.3%), and the proportion of unemployed participants was only about 10%. The percentage of participants having an average financial condition was the highest (76%), and the proportion of the five groups of quintile monthly family income was approximately equal (approximately 20%). The mean age was 40 (SD = 8.9), and there was no statistical difference between the three groups of polysubstance use. 

Methadone-treatment-related characteristics among patients are presented in Table 2. The mean MMT duration was 3.3 years (SD = 2.2), and the mean of the current MMT dose was 69.2 mg (SD = 37). The proportion of participants receiving MMT treatment at a private facility was nearly similar to those being treated in a public facility (49.4% and 50.6%, respectively). Approximately two-thirds of participants reported a history of injecting drug (63.8%). Approximately half of the patients reported optimal MMT adherence (43.3%), and there was no statistical difference between the three study groups. 

Table 3 illustrates the substance use patterns among MMT patients. In terms of the frequency of alcohol drinking, half of the participants had never drunk alcohol (46.6%), and about one-fourth of the participants drank alcohol every day (24.8%). More than two-thirds of respondents smoked on a regular basis (68.6%). Only 6% of MMT patients used illicit drugs (5.8%), and among them, 78.3% used them once per week. Craving was the reason for maintaining drug use for six patients (21.6%). Peer pressure was considered as the main reason for maintaining drug use (47.8%). 

Table 4 shows the factors that were associated with different types of substance abuse. In terms of alcohol drinking, participants who lived with a spouse/partner (OR = 2.01; 95% CI = 1.05–3.86) and were self-employed (OR = 2.33; 95% CI = 1.01–5.39) were more likely to drink alcohol. Smoking was a factor increasing the likelihood of drinking alcohol (OR = 3.06; 95% CI = 1.73–5.43), and participants drinking alcohol were more likely to smoke tobacco (OR = 3.11; 95% CI = 1.77–5.46). Compared with optimal adherence, MMT suboptimal adherence was associated with a higher likelihood of illicit drug usage (OR = 24.24; 95% CI = 3.1–189.8) as well as polysubstance use (Coef = 0.12; 95% CI = 0.01–0.22).

## 4. Discussion

This study enriches the current literature about the pattern of substance use among MMT patients in the developing country of Vietnam. In this study, we found a substantially high prevalence of polysubstance use in MMT patients. Moreover, the polysubstance use in our patients was driven by some critical factors such as medication adherence and service delivery models.

Our finding indicates that tobacco is the most common (81%) substance used among patients participating in the MMT program. This result is in line with previous reports which also found an extremely high level of cigarette use (the prevalence varies between 77% and 88%) in MMT patients [29,31]. Among middle-income countries, Vietnam has a high level of cigarette consumption [27]. It is evident that nicotine binds to the nicotine acetylcholine receptors in the central nervous system and stimulates dopamine release in the nucleus acumens, increasing euphoria and drug liking in smokers [32,33]. The literature suggests that patients use nicotine to relieve withdrawal symptoms of heroin including depression, restlessness, and irritability [32,34]. Besides, smoking is a leading cause of incidence in illicit drug users [35,36]. However, there has been little research on smoking behavior as well as smoking cessation in this population in Vietnam. Further intervention should integrate the MMT program with smoking cessation services to control cigarette use among MMT patients.

The second most commonly used substance is alcohol (54%). Our result is comparable to a 2014 study of five MMT patients in Dehong Prefecture in Yunnan Province, China, which found that 58.6% of the sample reported current alcohol drinking [37]. However, a 2013 multisite (including both urban and rural areas) study of MMT patients found alcohol usage among patients to be at 29.6%, which is lower than the proportion of our sample [14]. A possible explanation could be that our patients’ sample was taken from an urban area in Vietnam, which facilitates more convenient access to alcohol. 

Only 6% of our patients reported the use of illicit drugs, which included heroin, amphetamine, ecstasy, morphine, and methamphetamine. Craving was the second most reported reason for maintaining drug use (26.1%), and drug craving can be lowered by adjusting methadone doses. Moreover, patients with suboptimal MMT adherence were more likely to have concurrent illicit drug use. This finding is consistent with similar relationships observed in MMT patients in other countries [38,39]. We suggest that to increase the efficacy of MMT, treatment adherence by MMT patients can be closely monitored, which would require healthcare workers to provide personalized support to each patient. Peer pressure was the most commonly cited reason for illicit drug use, which is consistent with a study in another MMT patient group in Vietnam [28]. Thus, it is important for policymakers to initiate community-based education in combination with social and family support to help methadone patients reduce illicit drug consumption. 

Also, we found that patients receiving treatment in a public facility were associated with a lower likelihood of using alcohol and polysubstance use. The field of private healthcare service in Vietnam is nascent and was only formally recognized just over 20 years ago. The scale of private healthcare services is small compared with the public sector, and in general, information about its services is limited [40]. As of the time of this writing, the quality of private healthcare services is also poorly regulated, resulting in a large variance in the quality of healthcare delivery compared with the more centralized model of delivery of public healthcare [40]. Since MMT remains a new field in the healthcare system, there is only one private health clinic presently providing MMT (Dai Dong private health clinic) in northern Vietnam [41]. After the policies enacted in 2012 for provincial budgets, copayment by patients in all HIV-related services has been required to reduce the financial burden [42]. It is suggested that this user fee not only pay for the methadone drug but also be used as a means of domestic resources [43]. Based on our findings, we recommend that the government provide its support and expertise in helping the private health sector establish an efficient and effective MMT program. 

There were further interesting findings regarding factors associated with alcohol use in our MMT patients. Firstly, patients who live with their partner or spouse were more likely to use alcohol compared with those who live alone. We postulate that this could be due to family and peer influence, which is a strongly associated factor in substance use [29]. Concerning this, future health strategies could target patients’ family and social circle when psycho-educating and counseling them about the risks of substance usage. Secondly, self-employed patients were more likely to engage in alcohol usage compared with those who were unemployed. There could be a cultural explanation for this finding. In Vietnam, alcohol drinking has become a sociocultural norm, especially when connecting with colleagues and business clients for those who are employed [44]. Thirdly, smokers were more likely to drink alcohol, and vice versa (OR = 3.06; 3.11). This association was not surprising, as many studies have shown that both tobacco and alcohol abuse are often comorbid among MMT patients [17,18,45]. Both substances serve to decrease anxiety and depression symptoms (albeit temporarily, for as long as the substance is still in the blood) [46,47,48] commonly experienced in opioid withdrawal. The importance of implementing a robust MMT program with a strong emphasis on patients’ adherence cannot be understated, as optimal adherence to MMT can prevent opioid withdrawal symptoms such as anxiety and malaise [8].

To the best of our knowledge, this is the first study investigating the pattern of polysubstance use in Vietnamese MMT patients. Our study provides a basis for further research to build on our findings and expand the understanding of factors driving the behavior of polysubstance use in Vietnamese MMT patients. There are several limitations to this study. As this is a cross-sectional study, causality relationships cannot be drawn among the various factors that we studied. Further research should address this with a longitudinal study design. Convenience sampling was used, and this could lead to a sample which may not be representative of the population. Moreover, the self-reported nature of the questionnaire potentially makes the results subject to response bias and providing inaccurate data [49]. While MMT clinics conduct urine tests for patients on MMT, periodically or unexpectedly, this study did not collect data on urine drug tests for cross-validation with the self-reported data. In addition, future works on the topic of polysubstance use disorder may benefit from looking into the possible use of benzodiazepine among the sampled cohort, as benzodiazepine abuse has been found to be an issue for MMT patients in the United States due to inability of methadone to block benzodiazepine’s effects. The engagement of MMT patients in smoking and drinking can also be further assessed by exploring the quantity consumed instead of just frequency of use, as in our study. Furthermore, information on dropouts among study participants as well as the drop-out history of the respondents is recommended to be included in future studies to provide a comprehensive picture of MMT adherence. 

## 5. Conclusions

In conclusion, we found that tobacco and alcohol are the most common substances used by MMT patients in Vietnam. The importance of the implementation of a robust MMT program with a strong emphasis on patients’ adherence cannot be understated, as we found that optimal adherence to MMT was associated with reduced illicit substance use. Furthermore, a community-based educational and support program on substance use could be launched to effectively target the significant effect of peer pressure on substance use. Finally, future research should further analyze the pattern of polysubstance usage among MMT patients in a longitudinal study to gain better insights into how to develop a more effective MMT program among people who inject drugs.

## Figures and Tables

**Table 1:** Demographic characteristics of participants.

Characteristics	Polysubstance Use						Total		*p*-Value
	None		One Substance		Two or More Substances				
	*n*	%	*n*	%	*n*	%	*n*	%	
**Total**	48	12.2	153	38.7	194	49.1	395	100.0	
**Education**									
Under secondary school	8	16.7	26	17.0	32	16.5	66	16.7	0.09 *
Secondary school	23	47.9	101	66.0	113	58.3	237	60.0	
Above secondary school	17	35.4	26	17.0	49	25.3	92	23.3	
**Marital status**									
Single	11	22.9	28	18.3	28	14.4	67	17.0	0.64 *
Living with partner/spouse	34	70.8	115	75.2	155	79.9	304	77.0	
Divorced/widowed	3	6.3	10	6.5	11	5.7	24	6.1	
**Occupation**									
Unemployed	7	14.6	13	8.5	13	6.7	33	8.4	0.04 *
Self-employed	18	37.5	47	30.7	74	38.1	139	35.2	
Blue-collar worker/farmer	14	29.2	36	23.5	42	21.7	92	23.3	
Business	6	12.5	11	7.2	11	5.7	28	7.1	
Other	3	6.3	46	30.1	54	27.8	103	26.1	
**Family financial condition**									
Wealthy	0	0.0	2	1.3	9	4.6	11	2.8	0.16 *
Average	35	72.9	116	75.8	149	76.8	300	76.0	
Poor	13	27.1	35	22.9	36	18.6	84	21.3	
**Quintile monthly family income**									
Poorest	15	31.3	32	20.9	33	17.0	80	20.3	0.31 *
Poor	7	14.6	36	23.5	39	20.1	82	20.8	
Middle	11	22.9	26	17.0	44	22.7	81	20.5	
Rich	6	12.5	35	22.9	41	21.1	82	20.8	
Richest	9	18.8	24	15.7	37	19.1	70	17.7	
	**Mean**	**SD**	**Mean**	**SD**	**Mean**	**SD**	**Mean**	**SD**	***p*-Value**
**Age**	39.9	10.2	39.8	8.8	40.1	8.6	40	8.9	0.96 ^#^
**Monthly family income** (USD)	434.3	640.7	434.3	593.4	425.7	464.4	430	537.5	0.53 ^#^

* Chi-squared test, ^#^ Kruskal–Wallis test.

**Table 2:** Methadone-treatment-related characteristics.

Characteristics	Polysubstance Use						Total		*p*-Value
	None		One Substance		Two or More Substances				
	*n*	%	*n*	%	*n*	%	*n*	%	
**MMT model**									
Private facility	18	37.5	73	47.7	104	53.6	195	49.4	0.12 *
Public facility	30	62.5	80	52.3	90	46.4	200	50.6	
**Ever injected drugs**									
Yes	30	62.5	95	62.1	127	65.5	252	63.8	0.79 *
No	18	37.5	58	37.9	67	34.5	143	36.2	
**MMT adherence**									
Optimal adherence	21	43.8	73	47.7	77	39.7	171	43.3	0.33 *
Suboptimal adherence	27	56.3	80	52.3	117	60.3	224	56.7	
	**Mean**	**SD**	**Mean**	**SD**	**Mean**	**SD**	**Mean**	**SD**	***p*-Value**
**MMT duration** (years)	3.4	2.3	3.4	2.2	3.1	2.2	3.3	2.2	0.28 ^#^
**MMT dose** (mg)	65.7	36.9	73.8	38.9	66.4	35.2	69.2	37.0	0.05 ^#^
**Age at onset of drug use**	26.5	8.4	25.7	7.9	26	7.6	25.9	7.8	0.80 ^#^

* Chi-squared test, ^#^ Kruskal–Wallis test. Abbreviation: MMT—methadone maintenance treatment.

**Table 3:** Substance use patterns among MMT patients.

Characteristics	*n*	%
**Alcohol drinking frequency**		
None	184	46.6
Once per week	72	18.2
Every week	41	10.4
Everyday	98	24.8
**Smoking frequency**		
None	75	19.0
Once per week	20	5.1
Every week	29	7.3
Everyday	271	68.6
**Concurrent illicit drug use**	23	5.8
**Frequency of concurrent drug use** (n = 23)		
Once per week	18	78.3
Every week	1	4.4
Everyday	3	13.0
More than once a day	1	4.4
**Reason for concurrent drug use** (n = 23)		
Craving	6	26.1
Habit	5	21.7
Peer pressure influence	11	47.8
Other	1	4.4

**Table 4:** Factors associated with different substance uses.

Characteristics	n	%	Alcohol Drinking		Smoking		Concurrent Illicit Drug Use		Polysubstance Use	
			OR ^1^	95% CI ^2^	OR	95% CI	OR	95% CI	Coef.	95% CI
**Education**										
Less than secondary school	66	16.7			Ref.	-	Ref.	-		
Secondary school	237	60.0			1.24	0.60; 2.58	0.69	0.17; 2.86		
More than secondary	92	23.3			0.64	0.28; 1.49	1.97	0.46; 8.44		
**Marital status**										
Single	67	17.0	Ref.	-						
Living with partner/spouse	304	77.0	2.01 **	1.05; 3.86						
Divorced/widowed	24	6.1	0.99	0.35; 2.75						
**Occupation**										
Unemployed	33	8.4	Ref.	-	Ref.	-	Ref.	-	Ref.	-
Self-employed	139	35.2	2.33 **	1.01; 5.39	0.95	0.38; 2.36	0.31	0.06; 1.59	0.13	−0.11; 0.37
Blue-collar/farmer	92	23.3	1.33	0.56; 3.18	1.02	0.40; 2.63	1.25	0.25; 6.25	0.10	−0.16; 0.35
Business	28	7.1	0.59	0.19; 1.82	1.54	0.45; 5.27	2.20	0.38; 12.77	−0.01	−0.36; 0.34
Other	103	26.1	1.42	0.59; 3.39	4.38 ***	1.43; 13.42	0.88	0.19; 4.13	0.20	−0.04; 0.45
**Quintile monthly family income**										
Poorest	80	20.3							Ref.	-
Poor	82	20.8							0.12	−0.05; 0.29
Middle	81	20.5							0.14	−0.04; 0.32
Rich	82	20.8							0.13	−0.03; 0.30
Richest	70	17.7							0.13	−0.06; 0.32
**Age**	395	100.0	1.03 **	1.00; 1.06	0.96 ***	0.93; 0.99				
**MMT model**										
Private facility	195	49.4	Ref.	-			Ref.	-	Ref.	-
Public facility	200	50.6	0.60 **	0.39; 0.93			0.39 *	0.14; 1.06	−0.13 **	−0.23; −0.02
**Ever injected drugs**										
No	252	63.8	Ref.	-						
Yes	143	36.2	1.36	0.86; 2.14						
**MMT adherence**										
Optimal adherence	171	43.3	Ref.	-			Ref.	-	Ref.	-
Suboptimal adherence	224	56.7	1.35	0.87; 2.10			24.24 ***	3.10; 189.80	0.12 **	0.01; 0.22
**MMT dose** (mg)	395	100.0			1.00	0.99; 1.00				
**Age at onset of drug use**	395	100.0					0.90 **	0.82; 0.98		
**Alcohol drinking**										
No	184	46.6			Ref.	-				
Yes	211	53.4			3.11 ***	1.77; 5.46				
**Smoking**										
No	75	19.0	Ref.	-			Ref.	-		
Yes	320	81.0	3.06 ***	1.73; 5.43			3.22	0.64; 16.19		
*** *p* < 0.01, ** *p* < 0.05, * *p* < 0.1										

^1^ Odds ratio. ^2^ Confidence intervals.

## References

[1] Joint United Nations Program on HIV/AIDS (UNAIDS) (2014). Vietnam AIDS Response Progress Report.

[2] UNAIDS Country Factsheets—Vietnam 2017. https://www.unaids.org/en/regionscountries/countries/vietnam.

[3] Avert.org Funding for HIV and AIDS. https://www.avert.org/professionals/hiv-around-world/global-response/funding#footnote21_1clcn3t.

[4] NAC-National AIDS Commission (2002). Country Report on Follow-Up to the Declaration of Commitment on Hiv/Aids.

[5] Corsi K.F., Lehman W.K., Booth R.E. (2009). The effect of methadone maintenance on positive outcomes for opiate injection drug users. J. Subst. Abuse Treat..

[6] Jiang H., Han Y., Du J., Wu F., Zhang R., Zhang H., Wang J., Zhou Z., Hser Y.I., Zhao M. (2014). Factors associated with one year retention to methadone maintenance treatment program among patients with heroin dependence in China. Subst. Abuse Treat. Prev. Policy.

[7] Liu C., Liu P.L., Dong Q.L., Luo L., Xu J., Zhou W., Wang X. (2017). Social-demographic shift in drug users at the first-ever-methadone maintenance treatment in Wuhan, China. Sci. Rep..

[8] Nguyen T., Nguyen L.T., Pham M.D., Vu H.H., Mulvey K.P. (2012). Methadone maintenance therapy in Vietnam: An overview and scaling-up plan. Adv. Prev. Med..

[9] Mattick R.P., Breen C., Kimber J., Davoli M. (2009). Methadone maintenance therapy versus no opioid replacement therapy for opioid dependence. Cochrane Database Syst. Rev..

[10] Health A.G.D.o. (1995). The Principles of Methadone Maintenance Therapy.

[11] World Health Organization (2004). Substitution maintenance therapy in the management of opioid dependence and HIV/AIDS prevention. Who/Unodc/Unaids Position Paper.

[12] Viet Nam Authority of HIV/AIDS Control. Report on HIV/AIDS Prevention and Control in 2017 and Key Tasks in 2018. http://vaac.gov.vn/solieu/Detail/Bao-cao-cong-tac-phong-chong-HIV-AIDS-nam-2017-va-nhiem-vu-trong-tam-nam-2018.

[13] Giang L.M., Ngoc L.B., Hoang V.H., Mulvey K., Rawson R.A. (2013). Substance use disorders and HIV in Vietnam since Doi Moi (Renovation): An overview. J. Food Drug Anal..

[14] Connor J.P., Gullo M.J., White A., Kelly A.B. (2014). Polysubstance use: Diagnostic challenges, patterns of use and health. Curr. Opin. Psychiatry.

[15] Barocas J.A., Wang J., Marshall B.D.L., LaRochelle M.R., Bettano A., Bernson D., Beckwith C.G., Linas B.P., Walley A.Y. (2019). Sociodemographic factors and social determinants associated with toxicology confirmed polysubstance opioid-related deaths. Drug Alcohol Depend..

[16] Khoi D., Victor M., Rafat H. (2012). HIV Risks Among Injecting Drug Users in Vietnam: A Review of the Research Evidence. Curr. HIV Res..

[17] National Institute of Hygiene and Epidemiology (2014). An annual update on the HIV epidemic in Viet Nam. Viet Nam Authority of HIV/AIDS Control.

[18] Nguyen V.T., Scannapieco M. (2008). Drug abuse in Vietnam: A critical review of the literature and implications for future research. Addiction.

[19] Hainer R. Polysubstance Use and Stimulants: A Dangerous Fourth Wave in the Opioid Crisis. https://www.bmc.org/healthcity/population-health/polysubstance-use-dangerous-fourth-wave-opioid-crisis.

[20] Trujillo K.A., Smith M.L., Guaderrama M.M. (2011). Powerful behavioral interactions between methamphetamine and morphine. Pharm. Biochem. Behav..

[21] Richter K.P., Hamilton A.K., Hall S., Catley D., Cox L.S., Grobe J. (2007). Patterns of smoking and methadone dose in drug treatment patients. Exp. Clin. Psychopharmacol..

[22] Shadel W.G., Stein M.D., Anderson B.J., Herman D.S., Bishop S., Lassor J.A., Weinstock M., Anthony J.L., Niaura R. (2005). Correlates of motivation to quit smoking in methadone-maintained smokers enrolled in a smoking cessation trial. Addict. Behav..

[23] Teplin D., Raz B., Daiter J., Varenbut M., Plater-Zyberk C. (2007). Screening for alcohol use patterns among methadone maintenance patients. Am. J. Drug Alcohol Abuse.

[24] Makarenko I., Mazhnaya A., Marcus R., Pykalo I., Madden L., Filippovich S., Dvoriak S., Altice F.L. (2018). Concurrent drug injection during opioid agonist treatment among people who inject drugs in Ukraine. J. Subst. Abuse Treat..

[25] Maremmani I., Pani P.P., Mellini A., Pacini M., Marini G., Lovrecic M., Perugi G., Shinderman M. (2007). Alcohol and cocaine use and abuse among opioid addicts engaged in a methadone maintenance treatment program. J. Addict. Dis..

[26] Mason B., Kocsis J., Melia D., Khuri E., Sweeney J., Wells A., Borg L., Millman R., Kreek M., Kreek M. (1998). Psychiatric comorbidity in methadone maintained patients. J. Addict. Dis..

[27] Tran B.X., Ohinmaa A., Mills S., Duong A.T., Nguyen L.T., Jacobs P., Houston S. (2012). Multilevel predictors of concurrent opioid use during methadone maintenance treatment among drug users with HIV/AIDS. PLoS ONE.

[28] Do H.P., Nguyen L.H., Thi Nguyen N.P., Ngo C., Thi Nguyen H.L., Le G.T., Nguyen L.K., Nguyen C.T., Tran B.X., Le H.T. (2017). Factors associated with nicotine dependence during methadone maintenance treatment: Findings from a multisite survey in Vietnam. BMJ Open.

[29] Tran B.L., Boggiano V., Lan Thi Nguyen H., Hoang L., Van Nguyen H., Dinh Hoang C., Thi Le H., Dinh Tran T., Quan Le H., Latkin C. (2017). Concurrent drug use among methadone maintenance patients in mountainous areas in Northern Vietnam. BMJ Open.

[30] Dobler-Mikola A., Hattenschwiler J., Meili D., Beck T., Boni E., Modestin J. (2005). Patterns of heroin, cocaine, and alcohol abuse during long-term methadone maintenance treatment. J. Subst. Abuse Treat..

[31] Naji L., Dennis B.B., Bawor M., Plater C., Pare G., Worster A., Varenbut M., Daiter J., Marsh D.C., Desai D. (2016). A prospective study to investigate predictors of relapse among patients with opioid use disorder treated with methadone. Subst. Abuse Res. Treat..

[32] Feng N., Lin C., Hsieh J., Rou K., Li L. (2018). Family related factors and concurrent heroin use in methadone maintenance treatment in China. Subst. Use Misuse.

[33] Tran B.X., Nguyen L.H., Tran T.T., Latkin C.A. (2018). Social and structural barriers for adherence to methadone maintenance treatment among Vietnamese opioid dependence patients. PLoS ONE.

[34] Elfil M., Negida A. (2017). sampling methods in clinical research: An educational review. Emergency.

[35] Kelly P.J., Baker A.L., Deane F.P., Kay-Lambkin F.J., Bonevski B., Tregarthen J. (2012). Prevalence of smoking and other health risk factors in people attending residential substance abuse treatment. Drug Alcohol Rev..

[36] Williams J.M., Foulds J., Dwyer M., Order-Connors B., Springer M., Gadde P., Ziedonis D.M. (2005). The integration of tobacco dependence treatment and tobacco-free standards into residential addictions treatment in New Jersey. J. Subst. Abuse Treat..

[37] WHO (2010). Global Adult Tobacco Survey (GATS) in Viet Nam.

[38] Benowitz N.L. (2009). Pharmacology of nicotine: Addiction, smoking-induced disease, and therapeutics. Annu. Rev. Pharmacol. Toxicol..

[39] Nguyen N.P., Tran B.X., Hwang L.Y., Markham C.M., Swartz M.D., Phan H.T., Nong V.M., Nguyen C.T., Nguyen A.H., Latkin C.A. (2015). Prevalence of cigarette smoking and associated factors in a large sample of HIV-positive patients receiving antiretroviral therapy in Vietnam. PLoS ONE.

[40] Elkader A.K., Brands B., Selby P., Sproule B.A. (2009). Methadone-nicotine interactions in methadone maintenance treatment patients. J. Clin. Psychopharmacol..

[41] Liu L.Z., Tan L.X., Xu Y.M., Liu X.B., Chen W.C., Zhu J.H., Lu J., Zhong B.L. (2017). Significantly more intense pain in methadone-maintained patients who are addicted to nicotine. Oncotarget.

[42] Hurt R.D., Offord K.P., Croghan I.T., Gomez-Dahl L., Kottke T.E., Morse R.M., Melton L.J. (1996). Mortality following inpatient addictions treatment: Role of tobacco use in a community-based cohort. JAMA.

[43] Hurt R.D., Eberman K.M., Croghan I.T., Offord K.P., Davis L.J., Morse R.M., Palmen M.A., Bruce B.K. (1994). Nicotine dependence treatment during inpatient treatment for other addictions: A prospective intervention trial. Alcohol Clin. Exp. Res..

[44] Nyamathi A., Cohen A., Marfisee M., Shoptaw S., Greengold B., de Castro V., George D., Leake B. (2009). Correlates of alcohol use among methadone-maintained adults. Drug Alcohol Depend..

[45] Duan S., Jin Z., Liu X., Yang Y., Ye R., Tang R., Gao M., Ding Y., He N. (2017). Tobacco and alcohol use among drug users receiving methadone maintenance treatment: A cross-sectional study in a rural prefecture of Yunnan Province, Southwest China. BMJ Open.

[46] Haskew M., Wolff K., Dunn J., Bearn J. (2008). Patterns of adherence to oral methadone: Implications for prescribers. J. Subst. Abuse Treat..

[47] Nguyen M.P., Wilson A. (2017). How could private healthcare better contribute to healthcare coverage in Vietnam?. Int. J. Health Policy Manag..

[48] Perrine R., Caroline L., Laurent M., Julien C., Marion M., Fabienne M., Bruno S., Alain M., Patrizia M.C., Laurent K. (2014). Predictors of Non-adherence to Methadone Maintenance Treatment in Opioiddependent Individuals: Implications for Clinicians. Curr. Pharm. Des..

[49] Tran B.X., Le Nguyen Q., Nguyen L.H., Phan H.T.T., Le H.T., Tran T.D., Vu T.T.M., Latkin C.A. (2017). Expanding co-payment for methadone maintenance services in Vietnam: The importance of addressing health and socioeconomic inequalities. BMC Health Serv. Res..

